# Longitudinal development in the preterm thalamus and posterior white matter: MRI correlations between diffusion weighted imaging and T2 relaxometry

**DOI:** 10.1002/hbm.23188

**Published:** 2016-03-21

**Authors:** Andrew Melbourne, Zach Eaton‐Rosen, Eliza Orasanu, David Price, Alan Bainbridge, M. Jorge Cardoso, Giles S. Kendall, Nicola J. Robertson, Neil Marlow, Sebastien Ourselin

**Affiliations:** ^1^ Centre for Medical Image Computing (CMIC) University College London United Kingdom; ^2^ Medical Physics University College Hospital London United Kingdom; ^3^ Academic Neonatology EGA UCL Institute for Women's Health London United Kingdom

**Keywords:** neonate, connectivity, relaxometry, white matter, g‐ratio

## Abstract

Infants born prematurely are at increased risk of adverse neurodevelopmental outcome. The measurement of white matter tissue composition and structure can help predict functional performance. Specifically, measurements of myelination and indicators of myelination status in the preterm brain could be predictive of later neurological outcome. Quantitative imaging of myelin could thus serve to develop biomarkers for prognosis or therapeutic intervention; however, accurate estimation of myelin content is difficult. This work combines diffusion MRI and multi‐component T2 relaxation measurements in a group of 37 infants born very preterm and scanned between 27 and 58 weeks equivalent gestational age. Seven infants have longitudinal data at two time points that we analyze in detail. Our aim is to show that measurement of the myelin water fraction is achievable using widely available pulse sequences and state‐of‐the‐art algorithmic modeling of the MR imaging procedure and that a multi‐component fitting routine to multi‐shell diffusion weighted data can show differences in neurite density and local spatial arrangement in grey and white matter. Inference on the myelin water fraction allows us to demonstrate that the change in diffusion properties of the preterm *thalamus* is not solely due to myelination (that increase in myelin content accounts for about a third of the observed changes) whilst the decrease in the *posterior white matter* T2 has no significant component that is due to myelin water content. This work applies multi‐modal advanced quantitative neuroimaging to investigate changing tissue properties in the longitudinal setting. *Hum Brain Mapp 37:2479–2492, 2016*. © **The Authors Human Brain Mapping Published by Wiley Periodicals, Inc.**.

**Abbreviations**
EGA: Equivalent gestational age
EPG: Extended phase graph
MR: Magnetic resonance
MRI: Magnetic resonance imaging
MRS: Magnetic Resonance Spectroscopy
NAA: N‐Acetylaspartate
PWM: Posterior white matter

## INTRODUCTION

Very preterm birth (birth at less than 32 weeks completed gestational age) occurs at a time of rapid neurological development [Volpe, 2009]. During the period between 30 and 40 weeks gestation, the cerebral cortex of the brain changes from a smooth, unfolded state to one in which many secondary and some tertiary folds are present in advance of birth. In the developing white matter, processes leading to myelination progress in an established spatial pattern, ascending into the corticospinal tracts from as early as 30 weeks gestation and progressing from this region anterior and posterior over the first few months of life [Brody et al., 1987]. Infants born very preterm are at increased risk of adverse neurodevelopmental outcome [Costeloe et al., 2012] and this is believed to be related to delay or disruption to normal developmental processes during this crucial time. The reproducibility of the myelin developmental trajectory means that if myelin content can be measured, myelin location and quantity might be predictive of delays in the subsequent myelination process and thus of neurological developmental delay in infancy. A number of magnetic resonance (MR) based techniques have been used to infer myelin content including magnetization transfer [Stikov et al., 2011] and T2 relaxometry [Prasloski et al., 2012]. Diffusion Weighted MRI (DWI) is sensitive to local structure, but the short T2 of the myelin signal component means that DWI is generally non‐specific to myelin, although its presence will have an occult influence on parameters derived from a diffusion model. As a result any inference on myelin content using DWI alone must remain speculative. In this work we estimate the myelin water signal from multi‐echo multi‐component T2 relaxometry [Prasloski et al., 2012] and combine this with the intra‐axonal volume signal measured by a multi‐compartment DWI measurement [Alexander et al., 2010]; this imaging combination can be used to define a joint in vivo imaging biomarker that makes use of the structural sensitivity, but myelin inspecificity, of DWI with the high myelin specificity, but structural insensitivity of multi‐echo T2 relaxometry. Previous work has combined similar measurements in both quantitative [Melbourne et al., 2014a] and non‐quantitative [Stikov et al., 2011] frameworks to obtain the internal axon diameter to myelinated axon diameter g‐ratio [Chomiak and Hu, 2009]. Since this measurement has the potential to map the electrical properties of axons, it may be correlated with specific functional measurements. Magnetic Resonance Spectroscopy (MRS) studies have demonstrated changes in white matter metabolism with brain development including an increase in N‐Acetylaspartate (NAA) with progressing brain maturity, and a decrease in choline (Cho). The amino acid NAA is synthesized primarily in neuronal (including axonal) mitochondria and is therefore likely to have a relationship to cell energy turnover [Kreis et al., 2002; Moffett et al., 2007]. In normal brain development, Cho related species incorporate into macromolecules during myelination and become invisible to MRS, thus enhancing the observed NAA/Cho ratio. Results of neonatal spectroscopy have been linked to function and motor outcome at one year of age [Kendall et al., 2013].

During the period between 30 − 40 weeks gestation, short‐range associative connections increase in number and the white matter cellular composition is altered in advance of subsequent myelination. On magnetic resonance imaging (MRI), the absence of myelin is a major contributor to the inverted contrast, relative to the adult brain, seen on T1 and T2 weighted images and thus there is interest in using MR measurement of myelin as a biomarker of later neurological outcome. Some developmental changes can be observed on diffusion weighted MRI; for instance in grey matter the increasing cortical connectivity between 30 and 40 weeks gestational age reduces the observed diffusion anisotropy in a characteristic pattern [McKinstry et al., 2002], but this imaging technique is mostly insensitive to myelination since the T2 of proton spins bound into associated proteins is very short (<60 ms). As discussed in [Huppi and Dubois, 2006] changes to the diffusion tensor may be observed before the histological presence of myelin and so it is quite difficult to make statements about myelination without specific quantification. Furthermore, in the adult brain, only moderate correlations between diffusion parameters and multi‐component T2 were found by Maedler et al. [2008]. Single component relaxometry is thought to be a non‐specific indicator of myelin and myelination [Hagmann et al., 2009; He and Parikh, 2013] but as demonstrated by [Laule et al., 2006, 2008], a myelin water fraction *v*_mwf_ derived from multi‐component T2 relaxometry has a specific correlation with the results of histological staining. Nonetheless, changes to the cellular content of white matter have been used to infer the presence of myelin: during the preterm period; at term equivalent age and to follow myelination through infancy [Hagmann et al., 2009; Kulikova et al., in press; Nossin‐Manor et al., 2013; Partridge et al., 2004].

Recent studies have begun to investigate more quantitative measurements of neonatal brain development. Independently, Brown et al. [2014] and Pandit et al. [2014] investigated how the derived structural connectivity from diffusion weighted imaging differs with degree of prematurity and results of this type have begun to be linked to functional outcome at later ages [Ball et al., in press]. Recent work has moved away from the non‐quantitative framework of the diffusion tensor and begun to develop quantitative imaging parameters using biologically motivated model‐based diffusion‐weighted imaging and this has been applied to both white [Kunz et al., 2014] and grey matter [Eaton‐Rosen et al., 2015] properties of preterm brain tissue. Results of combinations of imaging modalities have been linked to demonstrate the relationship between cortical folding and diffusion MRI [Melbourne et al., 2014b] or to propose new imaging biomarkers [Melbourne et al., 2014a].

In this work we investigate how properties derived from both diffusion imaging and multi‐component T2 relaxometry change in the developing thalamo‐cortical system between 30 and 40 weeks equivalent gestational age (EGA) as assessed in cross‐sectional on a cohort of 37 infants which includes a sub‐cohort of longitudinal data for seven infants. We combine results from Diffusion weighted imaging, T2 relaxometry and proton spectroscopy to study longitudinal changes in the same infants at two time‐points. In contrast to previous work, here we attempt to combine only quantitative measurements such as the intra‐axonal and myelin‐water volumes in contrast to surrogate, measurements such as the fractional anisotropy or the single‐component T2.

We show how T2 values change in both the thalamus, the adjacent white matter and in the posterior white matter (PWM). In addition we show that the overall change in T2 can be attributed to an increase in myelination in the thalamus, but in the white matter this is not due to myelin but due to a reduction in free‐water content and increase in tissue volume. From DWI we show that the intra‐axonal volume fraction increases in the thalamus, but that this change can be explained by a combination of both myelin volume increase and axonal volume increase. We also present multi‐parametric results within common white‐matter regions of interest.

## METHOD

### Data

Imaging data were acquired for 42 preterm infants. Infants were excluded if they had abnormal cerebral ultrasound (2 infants), or if either the diffusion acquisition or the relaxometry acquisition was unusable due to patient movement (3 infants). Infant MRI was assessed using a white matter scoring system [Woodward et al., 2006]. No infants in this study were graded as having moderate or severe injury. The remaining 37 (10/27 M/F) preterm infants comprised 15 infants with data acquired at approximately 31 and 42 weeks EGA, four infants with data acquired at approximately 30 weeks EGA only and 18 infants with data acquired at approximately 40 weeks EGA only. Seven infants had usable longitudinal data (5/2 M/F). Imaging was carried out on a 3T Phillips Achieva. Imaging data was acquired without sedation in natural sleep, using a MR compatible incubator with neonatal specific head coil (http://www.lmt-medicalsystems.com). Informed parental consent was obtained for all infants and the study was approved by the local research ethics committee. Cohort information is summarized in Table 1.

**Table 1:** Selected demographic information for preterm cohort

Male/Female	10/27
Gestational age at birth	26.27 ± 2.10 weeks
EGA at early scan	31.02 ± 1.91 weeks
EGA at late scan	41.66 ± 4.28 weeks
Birthweight	844 ± 141g
Antenatal steroids	94%
Postnatal steroids	6%
Sepsis	13%
CLD/BPD (O_2_ at >36 weeks)	76%
Medically treated for NEC	33%

EGA, Equivalent Gestational Age; CLD, Chronic Lung Disease; BPD, Bronchopulmonary Dysplasia; NEC, Necrotising Enterocolitis.

Whole brain diffusion weighted imaging was acquired over 16 directions at a *b*‐value of 750 s.mm − 2 and 32 directions at *b* = 2,000 s.mm − 2 at resolution 1.75 × 1.75 × 2 mm^3^. Whole brain 32‐echo multi‐component quantitative T2 imaging was acquired at 1.2 × 1.2 × 3 mm^3^ resolution using a 2D GraSE acquisition at 12 ms TE and resampled into the diffusion imaging space. In addition, proton MRS was acquired using water suppressed Point Resolved Spectroscopy (PRESS; TR/TE 2,288/288 ms) with a 14 × 13 × 11 mm^3^ voxel in the left PWM. Spectra were analysed using the AMARES algorithm in the jMRUI spectroscopy package. Peak‐area ratios of Cho/total creatine (Cr), NAA/Cho, and NAA/Cr, were calculated. MRS acquisition information was used to construct an estimate of the MRS voxel position in DWI space, thus defining a PWM region of interest. Thalamus segmentations were carried out using the method described in Eaton‐Rosen et al. (2015) using a preterm specific segmentation algorithm [Melbourne et al., 2013a].

### Multi‐Component T2 Relaxometry

Single exponential fitting of multi‐echo or multi echo‐time (TE) data can be used to generate quantitative maps of T2 value [Hagmann et al., 2009]. We assume that the tissue composition can be described by a continuous (but computationally discrete and finite) set of compartments each with it's own associated T2 distribution undergoing exponential decay.

(1) $$S\left(n\right)=\int m\left(T2\right)exp\left(-\frac{nTE}{T2}\right)dT2$$

Of importance in this model is the detection of signal with T2 < 50 ms which is associated with a water signal that is closely interacting with myelin. This provides an indirect measurement of myelin content, termed the myelin water fraction, *v*_mwf_ defined as the sum of all component magnitudes, m(T2), with a T2 < 50 ms [Prasloski et al., 2012]. We also define a free‐water volume, *v*_iso_, defined as the sum of all component magnitudes, m(T2), with a T2 > 350 ms. In the case of the neonatal data used here, which uses a multiply refocused echo train, the imaging sequence is theoretically susceptible to B1‐inhomogeneity [Melbourne et al., 2013b]. Multi‐spin echo T2 decay generally assumes a train of perfect refocusing pulses that implies a perfectly homogenous B1 field and uniform flip angle *α*. In practice this condition is not met with the consequence that stimulated echoes are produced along the echo train. However, these may be modeled using the extended phase graph (EPG) algorithm [Prasloski et al., 2012] for n echoes in such a way that the local refocusing angle, can be estimated by simulating the history of previous imperfect refocusing pulses for *N*_c_ T2 components. This effect can distort estimates of the T2 distribution, particularly the short T2 components. Initial T2 component fitting was carried out using the EPG algorithm (EPG) to extract a short component from three T2 components to estimate the local applied flip angle α. We found that spatial homogeneity is high and flip angle remains above 90% of the applied angle. This weak inhomogeneity is not expected to distort a standard non‐negative least squares multi‐exponential fit [Prasloski et al., 2012].

### Multi‐Compartment Diffusion Weighted Imaging

We fit a multi‐ compartment signal model to the multi‐shell data using non‐linear least squares specifically to estimate an intra‐axonal volume fraction [Eaton‐Rosen et al., 2015; Zhang et al., 2012].

(6) $$S_{a}\left(b,x\right)=S_{a0}\;[v_{iso}^{\prime }e^{-bd_{iso}}+v_{in}^{\prime }\int f\left(n,\gamma \right)e^{-bd||(x.n)}d\Omega +v_{ex}^{\prime }e^{-bxD^{*}x}]$$

The signal model attributes the white matter signal measured by DWI to three compartments; an intra‐ axonal space *v*′_in_ and extra‐axonal space *v*′_ex_ and a free‐isotropic space *v*′_iso_ [Alexander et al., 2010]. Given the experimental *b*‐value, *b*, and gradient direction, ***x***, the signal from the intra‐axonal and extra‐axonal spaces is coupled by a specific distribution, *f*(***n*** *,γ*), which is assumed to represent axonal dispersion; formally a Watson distribution of oblateness γ, varying between 0, for highly oriented axons, up to 1 when there is no preferred structural orientation [Zhang et al., 2012]. A principal diffusion direction incorporated into the extra‐axonal diffusion tensor *D* ^∗^ can be defined by two angular parameters {*θ*, *φ*}. Lastly, *d*_ǁ_ and *d*_iso_ describe the parallel (to the principal diffuse direction) and isotropic diffusivities respectively.

### Estimating an *In‐Vivo* g‐Ratio

Both axon diameter and myelin diameter have an impact on the physical properties of nerve conduction. The ratio of internal axonal diameter to the total nerve diameter (axon + myelin) is a useful number that has theoretical relationships to axonal conduction velocity and energetic cost. This measurement is known from in vitro and histological studies of the electrical properties of axons [Chomiak and Hu, 2009]. Emergent approximations of this g‐ratio, can be measured using MRI [Melbourne et al., 2014a; Stikov et al., 2011].

We approximate *n* axons as long cylinders with an internal axon radius of *r*_in_ and an external myelin + axonal radius of r_out_. Assuming a cylindrical geometry, the intra‐axonal space is given by *v*_in_ = *n*2*πr* ^2^ *s* and the myelin volume by *v*_mwf_ = *n*2*π*( $r_{\text{out}}^{2}$ − $r_{\text{in}}^{2}$) s where s is a fixed axonal length. Taking the ratio of *v*_mwf_/***v*_in_** yields an expression for the bulk average g‐ratio, Γ (7) in terms of the myelin volume *v*_mwf_ and the intra‐axonal volume *v*_in_, capitalized to represent the bulk average nature of this measurement.

(7) $$\Gamma ={\left(\frac{v_{mwf}}{v_{in}}+1\right)}^{-0.5}$$

Using only DWI or multi‐component relaxometry is insufficient to estimate both *v*_in_ and *v*_mwf_. To reconcile these two modalities we make use of a four‐compartment tissue model [Alexander et al., 2010].

(8) $$S_{\text{total}}=v_{\text{mwf}}S1+v_{\text{in}}S2+v_{\text{ex}}S3+v_{\text{iso}}S4$$

The last three compartments of (8) are measurable using a multi‐compartment diffusion model [Zhang et al., 2012]. The model allows for the estimation of the signal from multiple compartments, specifically the intra‐axonal volume fraction associated with highly directional structure, *v*_in_. The intra‐axonal volume fraction associated with highly directional structure, *v*_in_. The remaining model compartment for S1 describes signal associated, in white matter, primarily with myelin and can be estimated by T2 relaxometry. Finally, because the diffusion signal model contains no signal from S1 it is necessary to multiply the estimates of *v*'_in_, *v*'_ex_ and *v*'_iso_ from the diffusion measurement (6) by (1 − *v*_mwf_) from (5) and hence, *v*_in_ = *v*'_in_(1 − *v*_mwf_). If the change in a diffusion imaging parameter such as v'_in_ was purely due to a change in local quantity of myelin (or at least DWI invisible structure), then the new value could be estimated from the old value and the correction factor of 1 − *v*_mwf_.

### Regions of Interest

Thalamus regions of interest are defined using a preterm specific segmentation routine [Melbourne et al., 2013a]. PWM regions of interest are defined by the spectroscopy voxel placement. PWM‐tract based regions of interest are defined between the thalamus ROI and the posterior white matter ROI. For this purpose we use the multi‐directional ball and stick model and probabilistic tractography method described in Behrens et al. [2007]. Connectivity distributions are defined between the thalamus as a seed mask and the posterior white matter MRS voxel as the target mask. The distributions are then used to form weighted parameter estimates for this regions of interest. Furthermore, a thalamus‐tract ROI is defined by the number of times each voxel within the thalamus seed mask is able to reach the PWM target region. This implicitly weights the thalamus ROI by how easily each voxel within it can reach the PWM ROI. For each infant, longitudinal progression in the values of *v*_in_, *v*_mwf_, Γ and T2 can then be observed in space as well as between (equivalent) gestational ages.

Thus for each infant we are able to show results that have been weighted for both: (1) the thalamus, weighted by how easy it is for a tractography algorithm beginning in a particular voxel within the thalamus mask to reach any part of the MRS voxel (as a percentage of the number of trials); and (2) the PWM region that is passed through by the tractography algorithm between the thalamus and the MRS voxel, weighted by how often each voxel is traversed. We adopt this pragmatic definition of what is meant by the results of a tractography algorithm to avoid biological interpretation fallacies when using notions of fibre connectivity and fibre integrity [Jones et al., 2013].

**Figure 1:**
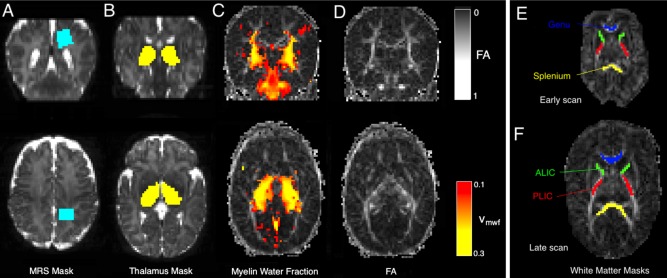
Example imaging data for single term‐infant. (**a**) T2‐weighted image overlaid with MRS voxel position, (**b**) T2‐ weighted image overlaid with thalamic segmentation, (**c**) Estimated regions of myelin water fraction > 0.01 overlaid on FA map and (**d**) FA map. Example white matter regions of interest (PLIC, ALIC, genu and splenium) are shown for (**e**) an early scan and (**f**) a term‐equivalent age scan. [Color figure can be viewed in the online issue, which is available at http://wileyonlinelibrary.com.]

Cross‐sectional ROIs are manually delineated for each infant in the four regions: the bilateral posterior and anterior limbs of the internal capsule (PLIC and ALIC respectively) and the genu and splenium of the corpus callosum (Fig. 1e,f). We additionally investigate manually placed bilateral regions of interest in the anterior white matter for comparison with the spectroscopic voxel in the PWM.

### Statistical Analysis

Two sample *t*‐tests were used between imaging parameters. Results are reported to be significant if the corresponding statistical *P*‐value is less than 0.05. Results that are corrected for age at birth or age at scan are reported from partial linear correlation coefficients with statistical significance if the (equivalent) *P*‐value is less than 0.05. Parameter rates of change are estimated from linear fits to the parametric data and EGA. Parameter rates of change can be compared through the use of the Fisher z‐transform.

## RESULTS

Figure 1 shows white matter spectroscopy and thalamic masks and MWF maps for a representative term age infant. Masks defined by the spectroscopy voxel and of the thalamus using the data and method of Eaton‐Rosen et al. [2015] are shown overlaid on T2‐weighted images. The estimated MWF for this infant is shown overlaid on T2‐weighted and FA maps from the diffusion imaging. Thalamic and spectroscopy voxel masks are used as endpoints of tractography.

Results are divided into four sections analysing: parameter changes in the preterm thalamus; parameter changes in the PWM; parameter changes along the diffusion pathway connecting these two regions and finally; results from the entire, and larger, cross‐sectional cohort.

### Longitudinal Diffusion and Myelin Water Fraction Changes in the Preterm Thalamus

Imaging parameter estimates for all longitudinal infants are shown in Table 2. Thalamic T2 is shown to decrease from an average of 203 ± 7 ms to 181 ± 7 ms (correlation coefficient *ρ* = −0.84, *P* < 0.001). This is associated with a short T2 component that increases its volume fraction from 0.05 ± 0.02 to 0.11 ± 0.02 (*ρ* = 0.87, *P* < 0.0001). Adjusting the diffusion MRI measured intra‐axonal change with knowledge of the myelination change using the correction strategy in Section 3.3 suggests that myelin alone is insufficient to explain the longitudinal increase in *v*_in_ in the preterm thalamus, accounting for less than a third (0.27 ± 0.06) of the observed change. Additionally, the estimated voxel average g‐ratio, Γ, is seen to decrease with increasing EGA.

**Table 2:** Thalamic parameter values for multi‐modal MRI. Data is shown at birth (b), early scan (*P*) and late scan (*t*)

Infant	EGA (weeks)	*v′*_in_	*v*_mwf_	Corrected *v*_in_	*g*‐ratio	T2 (ms)
1b	26.14					
1p	33.14	0.189 (0.0032)	0.0586 (0.0025)	0.178 (0.003)	0.874 (0.005)	203 (2)
1t	40.14	0.241 (0.0026)	0.115 (0.0027)	0.213 (0.0024)	0.823 (0.0039)	177 (1.9)
2b	25.14					
2p	31.43	0.188 (0.0028)	0.0678 (0.002)	0.175 (0.0026)	0.857 (0.004)	197 (1.1)
2t	42	0.245 (0.0021)	0.132 (0.0028)	0.213 (0.0019)	0.806 (0.0038)	175 (1.5)
3b	25.14					
3p	31	0.175 (0.0026)	0.0289 (0.0011)	0.17 (0.0025)	0.926 (0.0029)	204 (1.2)
3t	42	0.242 (0.0019)	0.11 (0.0024)	0.215 (0.0018)	0.829 (0.0034)	172 (1.7)
4b	27.14					
4p	30.86	0.166 (0.0039)	0.0353 (0.0012)	0.16 (0.0037)	0.908 (0.0034)	208 (0.76)
4t	46.29	0.258 (0.0023)	0.115 (0.0026)	0.228 (0.0021)	0.832 (0.0035)	178 (2.3)
5b	27.14					
5p	29.86	0.154 (0.0026)	0.045 (0.0019)	0.147 (0.0025)	0.88 (0.0047)	215 (1.3)
5t	46.29	0.264 (0.0025)	0.109 (0.0034)	0.235 (0.0024)	0.842 (0.0046)	186 (2.8)
6b	26.29					
6p	31.86	0.183 (0.0037)	0.0712 (0.0023)	0.17 (0.0034)	0.849 (0.0049)	194 (1.7)
6t	40.29	0.216 (0.0026)	0.0899 (0.0033)	0.197 (0.0025)	0.84 (0.0052)	192 (2.8)
7b	26.29					
7p	34.57	0.191 (0.0037)	0.0394 (0.0016)	0.183 (0.0035)	0.911 (0.0037)	200 (1.4)
7t	40.29	0.248 (0.0022)	0.0846 (0.0026)	0.227 (0.0021)	0.863 (0.0038)	185 (2.4)

Confidence intervals on the mean are shown in parenthesis. Errors for *v*_in_ and Γ are estimated using the general formula for propagation of uncertainty. Corrected *v*_in_ values are shown after using the correction strategy described in Section 3.3.

### Longitudinal Diffusion and Myelin Water Fraction Changes in the Preterm Posterior White Matter

Table 3 contains imaging parameter values for the MRS region of interest defined in the PWM of each infant. T2 values are seen to fall over this approximately 10‐week gestational period (299 ± 11ms to 205 ± 93 ms) whilst NAA/Cho increases (0.40 ± 0.09 to 1.04 ± 0.17) and Cho/Cr decreases (2.79 ± 0.16 to 1.80 ± 0.34).

**Table 3:** PWM parameter values for multi‐modal MRI shown for v'_in_ estimated from diffusion MRI, T2 in value and MRS NAA/Cho and Cho/Cr values

Infant	EGA (weeks)	*v′*_in_	*v′*_mwf_	Corrected *v*_in_	T2 (ms)	NAA/Cho	Cho/Cr
1b	26.14						
1p	33.14	0.083 (0.073)	0.0164 (0.004)	0.0817 (0.072)	283 (42)	0.475	2.73
1t	40.14	0.116 (0.073)	0.0221 (0.018)	0.113 (0.072)	277 (42)	−	−
2b	25.14						
2p	31.43	0.115 (0.15)	0.0173 (0.005)	0.113 (0.14)	292 (40)	0.36	2.77
2t	42	0.135 (0.052)	0.0177 (0.02)	0.133 (0.051)	222 (27)	1.24	1.61
3b	25.14						
3p	31	0.095 (0.098)	0.0182 (0.008)	0.0935 (0.096)	290 (38)	0.475	2.79
3t	42	0.098 (0.053)	0.0376 (0.034)	0.094 (0.051)	243 (35)	1.06	1.5
4b	27.14						
4p	30.86	0.087 (0.075)	0.017 (0.006)	0.0854 (0.074)	305 (45)	0.445	2.58
4t	46.29	−	−	−	−	−	−
5b	27.14						
5p	29.86	0.067 (0.065)	0.0165 (0.004)	0.0659 (0.063)	316 (49)	0.334	2.69
5t	46.29	0.166 (0.045)	0.0253 (0.026)	0.162 (0.044)	209 (30)	1.04	1.86
6b	26.29						
6p	31.86	0.07 (0.087)	0.0169 (0.004)	0.0692 (0.085)	307 (36)	0.467	2.88
6t	40.29	0.09 (0.073)	0.0175 (0.007)	0.0887 (0.071)	242 (51)	−	−
7b	26.29						
7p	34.57	0.057 (0.06)	0.0176 (0.004)	0.056 (0.059)	302 (43)	0.251	3.08
7t	40.29	0.095 (0.068)	0.016 (0.008)	0.0933 (0.067)	240 (48)	0.828	2.25

Data is shown at birth (b), early scan (*P*) and late scan (*t*). Confidence intervals on the mean are shown in parenthesis. Term equivalent age spectroscopy data for infant 4 is unavailable. Corrected *v*_in_ values are shown after using the correction strategy described in Section 3.3.

### Longitudinal Diffusion and Myelin Water Fraction Changes in the Thamalo‐Posterior White Matter Region

**Figure 2:**
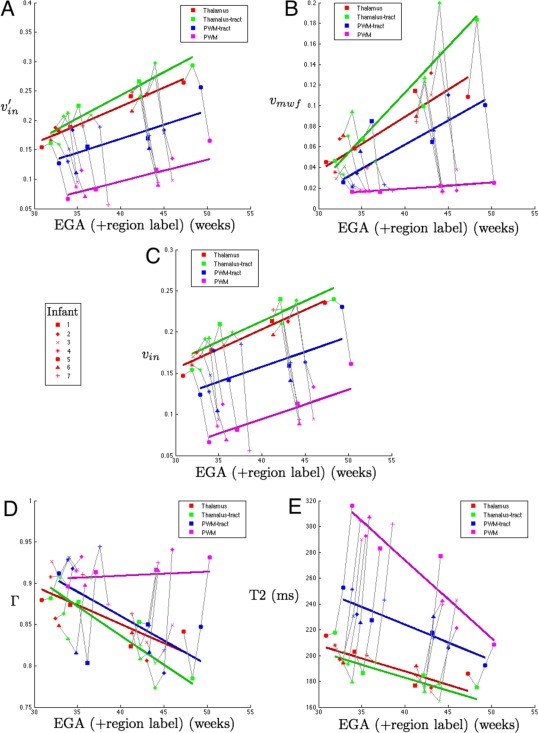
Combined results for individual infants showing spatiotemporal parametric changes estimated over: in Red) the entire thalamus (Thalamus); Green) thalamic voxels weighted by how often tracts appear to reach the MRS voxel (Thalamus‐tract); Blue) white matter voxels representing the most commonly found paths from the thalamus to the posterior white matter (PWM‐tract); and Magenta) average values in the MRS voxel (PWM) and (**a**) *v*′_in_ (uncorrected) (**b**) *v*_mwf_ **c**) *v*_in_ (corrected v′_in_) (**d**) g‐ratio (Γ) (**e**) T2. Results for Thalamus‐tract, PWM‐tract and PWM are displayed offset by 1, 2 and 3 in weeks respectively for clarity. [Color figure can be viewed in the online issue, which is available at http://wileyonlinelibrary.com.]

Combined results for each infant with longitudinal data are shown in Figure 2. For each infant we show results from the entire thalamus, and PWM (see Tables I and II), but additionally show parameter values that have been weighted for the thalamus and the PWM regions that are passed through by the tractography algorithm. For each infant, longitudinal progression in the values of *v*_in_, *v*_mwf_, Γ and T2 can be observed in space as well as between (equivalent) gestational ages.

Figure 2 illustrates the spatio‐temporal changes for this group of seven infants. Each infant has two time‐points each with four data points. Datapoints from the same scan are connected by solid grey lines. The marker style of these lines indicates the infant number (see figure legend) and is the same at both early and late scans. The first datapoint for each infant is located at the EGA at the time of the scan; subsequent connected points are spatially separated by 1 week to aid visualization. Solid colored lines show trends in the parameter values in each region with time across the cohort. Separation in tissue properties by location is shown across these seven infants: comparing the values found in the unweighted and tract‐weighted thalamus masks (see Table 2) shows higher values of *v*_in_ and *v*_mwf_ in the weighted thalamus measurement and additionally those regions have the lowest values of g‐ratio and T2. The lowest T2 values are estimated within the thalamus region, with little difference between the results of the weighted and unweighted thalamus mask. In the thalamus, the highest values for *v*_mwf_ are seen using the weighted mask suggesting that the regions of highest *v*_in_, (and also directionality, measured by the tractography) are associated with those regions that are estimated to contain the most myelin water. The highest values for T2 are found in the un‐weighted spectroscopy voxel, with substantially lower values in the diffusion‐derived tract system passing between thalamus and spectroscopy voxel. The strong fall in T2 in the PWM voxel is accompanied by an increase in *v*_in_ as measured by the DWI model and little change in the amount of myelin estimated in this region.

### Cross‐Sectional Parametric Correlations in Preterm White Matter

**Figure 3:**
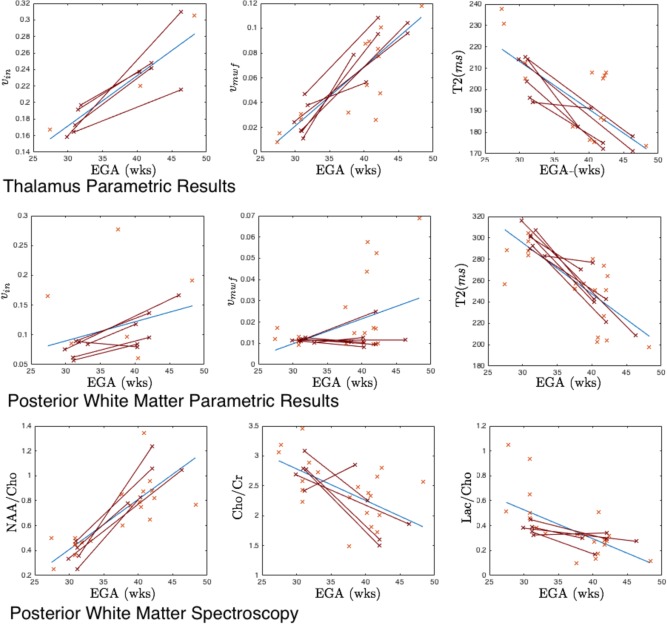
Cross sectional changes in *v*_in_, T2 and spectroscopy. Figures (**a**–**c**) thalamus parametric results for intra‐axonal volume fraction *v*_in_, myelin water fraction *v*_mwf_ and free water volume *v*_iso_ respectively; (**d**−**f**) PWM parametric results defined in the spectroscopy voxel ROI for *v*_in_, myelin water fraction *v*_mwf_, Tissue T2; (**i**–**k**) spectroscopy ratios NAA/Cho, Cho/Cr and Lac/Cho respectively. Cross‐sectional data are shown in blue, with blue trend line. Longitudinal data are shown in red, connected by red lines. [Color figure can be viewed in the online issue, which is available at http://wileyonlinelibrary.com.]

**Figure 4:**
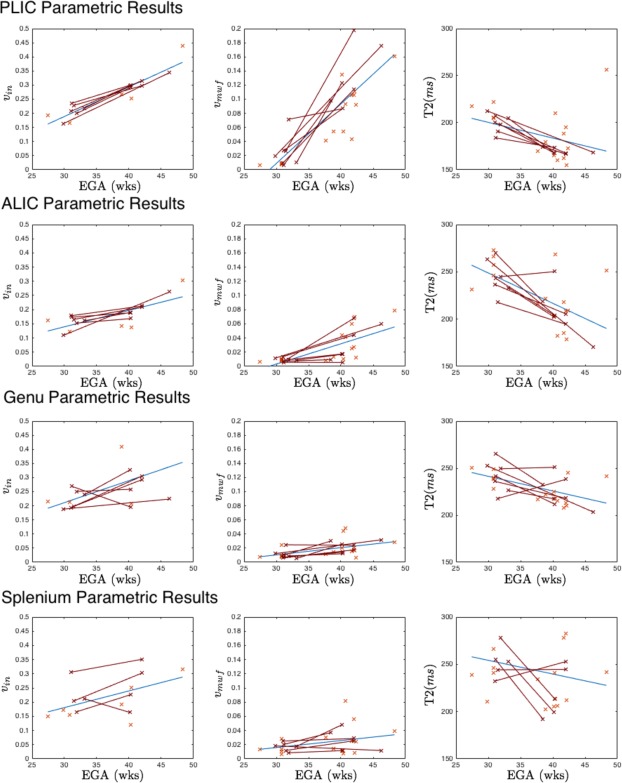
Cross sectional correlations between T2 relaxometry and spectroscopy for (**a**) T2 against NAA/Cho ratio, (**b**) vtissue against NAA/Cho ratio and (**c**) *v*_iso_ against NAA/Cho ratio. Cross‐sectional data are shown in blue, with blue trend line. Longitudinal data are shown in red, connected by red lines. [Color figure can be viewed in the online issue, which is available at http://wileyonlinelibrary.com.]

This section contains results for the larger cross‐sectional cohort of infants, including those with longitudinal data. Figure 3 shows trends in the parameter values for this larger group. Cross sectional trend lines are shown in blue, whilst longitudinal results are shown connected with red lines. In the thalamus (Fig. 4a–c), tissue T2 is seen to decrease at a rate of 2.24 ms/week (*r* = 0.76, *P* < 1 × 10^− 7^), *v*_mwf_ increases at a rate of 0.48%/week (*r* = 0.86, *P* < 1 × 10^−7^). In the PWM (Fig. 4d–h), tissue T2 is seen to decrease at a rate of 5 ms/week (*r* = 0.80, *P* < 1 × 10^−7^), *v*_mwf_ increases at a rate of 0.1%/week (*r* = 0.44, *P* = 0.011), *v*_iso_ decreases at a rate of 2.7%/week (*r* = 0.87, *P* < 1 × 10^−10^) and *v*_tissue_ increases at a rate of 2.6%/week (*r* = 0.87, *P* < 1 × 10^−9^).

Figure 4 shows changes in *v*_in_,. *v*_mwf_ and T2 in four central white matter regions. The top row of Figure 4 shows results for the PLIC, showing trends for increasing *v*_in_ (*r* = 0.93 *P* < 1 × 10^−7^) and *v*_mwf_ (*r* = 0.86, *P* < 1 × 10^−8^) and decreasing T2 (*r* = −0.41, *P* = 0.026) in both cross‐section and for the longitudinal infants with growth rates of 1%/week, 0.8%/week and −1.67 ms/week respectively. *v*_mwf_ values in the PLIC are quite high, commensurate with earlier myelination in this region and in contrast to what is expected in the ALIC (Fig. 4, second row) which has slower measured increases in *v*_in_ (0.5%/week, *r* = 0.75 *P* < 1 × 10^−4^) and *v*_mwf_ (0.2%/week, *r* = 0.72 *P* < 1 × 10^−5^). Although measurements of both *v*_in_ and *v*_mwf_ are significantly lower in the ALIC than the PLIC, the difference in *growth rate* is found to be significant only for *v*_in_ but not for *v*_mwf_.

In the corpus callosum (Fig. 4, third and fourth rows), results for the genu and splenium are found to be quite similar. Both regions have quite low measured values of *v*_mwf_ and comparable values of *v*_in_. Both *v*_in_ and *v*_mwf_ are found to be increasing in the genu (*v*_in_: 0.8%/week *r* = 0.58, *P* = 0.015) (*v*_mwf_: 0.1%/week *r* = 0.52, *P* = 0.004), but increases do not reach significance in the splenium (*v*_in_ *P* = 0.06, *v*_mwf_ *P* = 0.09). T2 values in both regions are falling (−1.56 ms/week (genu) vs −1.35 ms/week (splenium)), but this only reaches significance in the genu (*r* = 0.55, *P* = 0.002) compared to the splenium (*r* = 0.19, *P* = 0.34).

**Figure 5:**
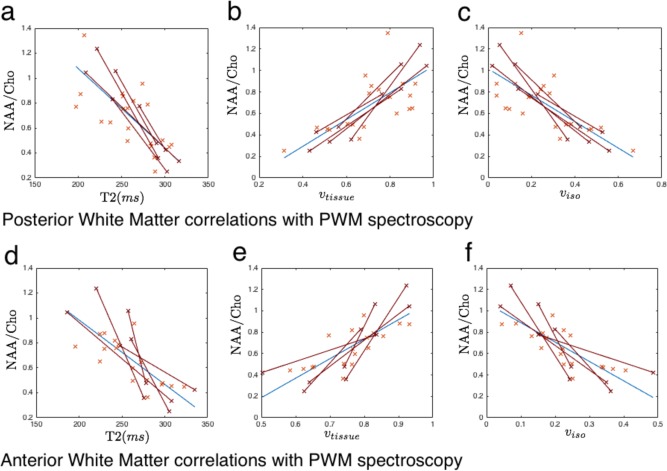
Cross sectional correlations between white matter T2 relaxometry and spectroscopy for top row: (**a**) PWM T2 against PWM NAA/Cho ratio, (**b**) PWM vtissue against PWM NAA/Cho ratio and (**c**) PWM *v*_iso_ against PWM NAA/Cho ratio and bottom row: (a) AWM T2 against PWM NAA/Cho ratio, (b) AWM vtissue against PWM NAA/Cho ratio and (c) AWM *v*_iso_ against PWM NAA/Cho ratio Cross‐sectional data are shown in blue, with blue trend line. Longitudinal data are shown in red, connected by red lines. [Color figure can be viewed in the online issue, which is available at http://wileyonlinelibrary.com.]

Figure 5 investigates the relationship between posterior and anterior white matter T2 and PWM spectroscopy. There is a high correlation between PWM NAA/Cho ratio and PWM T2 (*r* = 0.79, *P* < 1 × 10^−6^) that, by comparison with the results in Figure 3, could be related to a decrease in free‐water volume fraction and replacement by structured, but unmyelinated tissue, with shorter T2. Correcting for EGA reduces the correlation between NAA/Cho ratio and PWM T2 (*r* = 0.49, *P* = 0.008), but does not remove the significant interaction entirely suggesting a link between PWM T2 and PWM NAA/Cho that is independent of gestational age. Correlations between PWM NAA/Cho and v_tissue_ and *v*_iso_ are also significant correcting for EGA (*r* = 0.43, *P* < 0.02 and *r* = 0.44, *P* < 0.019 respectively). Comparison with tissue properties in the anterior white matter reveals similar, but less strong trends, the correlation between AWM T2 and PWM NAA/Cho is strongly significant (*r* = 0.76, *P* < 1 × 10^−6^) falling to *r* = 0.35 (*P* = 0.08 – no longer significant) when correcting for EGA. Although uncorrected trends between NAA/Cho and T2 in PWM and AWM are not significantly different from one another using a Fisher transform, correcting for gestational age dependence reveals that the correlation is much weaker between AWM T2 and PWM NAA/Cho than for PWM T2 and PWM NAA/Cho. This result is commensurate with differential rates of tissue development in the anterior and posterior brain once the general effect of gestational age is removed.

## DISCUSSION

In this work we have investigated how properties derived from both diffusion imaging and multi‐ component T2 relaxometry change in the developing thalami‐cortical system between 27 and 58 weeks EGA as assessed in cross‐sectional on a cohort of 37 infants which includes a sub‐cohort of longitudinal data for seven infants. We have combined results from Diffusion weighted imaging, T2 relaxometry and proton spectroscopy to study longitudinal changes in the same infants at two time‐points. In contrast to previous work, here we combined only quantitative measurements such as the intra‐axonal and myelin‐water volumes in contrast to surrogate, measurements such as the fractional anisotropy or the single‐component T2.

The imaging data acquired on this cohort has shown how MR measurable tissue properties vary longitudinally over the preterm period. Data from the diffusion imaging suggests that the intra‐axonal volume fraction increases in both the thalamus and the PWM over this period, commensurate with previous work [Eaton‐Rosen et al., 2015; Kunz et al., 2014]. Using multi‐compartment T2 relaxometry has been used to estimate the contribution to this change from myelin by measuring an increase in the myelin water fraction in both these regions; notably the increase in the thalamus is of greater magnitude. We use the myelin water measurement to correct the volume fractions measured from the diffusion imaging. This is because DWI is non‐specific to myelination [Maedler et al., 2008] and increases in intra‐axonal volume may be due to either increased myelin content or an increase in axon caliber or number. The result shows that increasing myelin content in the thalamus accounts for about one third of the change in axonal volume fraction measured solely from DWI. Additionally, the changes seen in the spectroscopy ratios; an increase in NAA/Cho and decrease in Cho/Cr are comparable to 1.5T values, although here the results are longitudinal not cross‐sectional [Kendall et al., 2013; Kreis et al., 2002]. The correlation between T2 and NAA/Cho that persists after correction for EGA suggests a synergy in the relationship between measured tissue structure and function.

The multi‐modal data presented in this work has allowed some of the confounding factors related to mono‐modal neonatal data to be explored. Of note, the rapid decrease in white matter T2 value is associated with a decrease in free water and an increase of tissue volume fraction. In the PWM, the contribution of measured myelin water is quite minor, and we tentatively attribute the dominant change to (unmyelinated) axonal and glial proliferation supported by the T2 relaxometry and spectroscopy results in Figure 3. The observed structural change in DWI and T2 relaxometry associated with increase NAA/Cho ratio that could imply an increase in the volume of functional tissue. This result is supported by the comparison of the PWM spectroscopy values with anterior white matter structural properties where the results suggest that once the influence of EGA is removed, more subtle differences in developmental trajectory between AWM and PWM can be observed. Conversely in the thalamus, the increase in intra‐axonal volume fraction more strongly related to an increase in measured myelin water as suggested in Eaton‐Rosen et al. [2015], but this does not entirely explain the tissue changes observed. About two‐thirds of this tissue structural change must be the result of other tissue composition changes contributing to increased apparent intra‐axonal volume fraction (and thus FA) in this region. In addition to the thalamus, we have shown how the PWM section of the thalamo‐cortical system is developing in the seven infants with longitudinal data. This data quantifies our general observations of tissue parameter change to specific individual cases and we are able to infer increases in thalamic myelin water fraction, changes to the white matter fiber structure and also infer how this correlates to functional spectroscopic data in the region to which it is connected.

Results in the white matter PLIC and ALIC show differentiation in axonal and myelin density with high *v*_mwf_ values in the posterior limb and much lower values in the ALIC despite relatively similar values of *v*_in_. Differential axonal and myelin density changes in these regions may make them good markers of future developmental changes, although the direct *v*_in_ measurements should be corrected by (1− *v*_mwf_) to remove the contribution of the myelin water space to the axonal density measurement. In the corpus callosum imaging properties related to axonal density, myelin density and T2 change in tandem and rates of change in these measurements are not distinguishable. Although some authors have found differences in imaging parameters between the genu and splenium [Partridge, 2004], the size of the cohort used in our work is quite low and the baseline of measurements quite narrow compared to other larger scale cross‐sectional studies.

It is possible that over the EGAs that we are measuring, that there is either a normalization of imaging parameters towards term equivalent age, or an otherwise variable rate of change [Ball et al., 2013]. The results in Figures 3 and 4 do not support this observation, and appear to suggest that the opposite may be true especially for those areas that are beginning to myelinate over this late gestational period. This is suggested by the *v*_mwf_ results in the genu and splenium in Figure 4 where the parameter variability increases. Figure 3 further suggests that the variability on the cross‐sectional measurements increases with gestational age. Over the longer‐term (the first year of life) parameter variability should reduce and stabilise, but in the period we are measuring there is rapid change in tissue volume and composition. Interestingly the individual parameter trajectories of the longitudinal infants are quite harmonious relative to one another. The results in Figures 3 and 4, and the limitation of only two timepoints for some subjects makes it difficult to infer variable or non‐linear parameter trajectories.

The main limitation of this work is the low number of infants with longitudinal data, although the number of infants with a single scan is much higher. Obtaining repeated MR acquisitions on this vulnerable cohort is quite challenging and is confounded with a relatively low initial number of extremely preterm infants suitable for scanning. However, data of this type is fundamental to understanding brain maturation and the effect of preterm birth on brain maturation. Ideally, multiple time‐points would be acquired on each infant over his late‐gestational period but this is not possible. The variation in the range of EGAs at scan is also an unavoidable limitation of our work. Some longitudinal imaging timepoints are more widely separated than others and it is unclear what the effect of this will be on our description of longitudinal parameter changes. The cross‐sectional data in our work appear well supported by linear relationships, although there is some evidence that parameter changes over this range of EGAs may not be linear [Ball et al., 2013]. With the data that we have, it is difficult with two longitudinal imaging time points to support an alternative model, and it is not clear how models applied to cross‐sectional data should really be applied in the longitudinal setting. However, the underlying results that suggest an increase in both thalamus axonal density and thalamus myelin density are unlikely to be altered. Additionally, a sub‐thalamic parcellation may reveal more interesting variation in axonal and myelin density within different thalamic nuclei. Although the combination of DWI and T2 relaxometry makes many assumptions about the image formation process (this is also true of the individual modalities too), the combination of these data does allow new information to be obtained. Importantly, measurement of the g‐ratio may, in future, be linked speculatively with simple physical models to predict the effect of change in myelin thickness on conduction velocity and energetic efficiency [Chomiak and Hu, 2009; Melbourne et al., 2014a]. The analysis of spectroscopy data could be bolstered by absolute quantification of spectroscopy peaks (although this would lengthen the neonatal acquisition). This would enable hypotheses to be tested about how the changes to white matter regions are due to axonal proliferation, linked to increasing NAA, or to myelination, since choline is a possible marker of the status of myelination.

Future work will use this multi‐modal approach to make predictions about functional development in preterm children. This is plausible since there is a well‐defined sequence of myelination from the PLIC outward [Brody et al., 1987], and delays to this might predict corresponding delays in functional progression of motor, language and executive function as the brain increases functional electrical energetic efficiency. Irrespective of the combination of measurements in this work, the acquisition of widely standard multi‐shell DWI and multi‐echo T2 imaging within clinically feasible time frames is important and will stimulate the generation of novel predictive structural biomarkers with a tangible physical link to neuronal function.

One of the main contributions of this work is the combination of results from a number of notionally quantitative imaging modalities. Investigation of mono‐modal properties can only reveal so much about the developing brain; metaphorically a single modality remains only a single piece of the neurodevelopment jigsaw. In isolation, imaging modalities can only be so informative, only by inspecting all the pieces in the puzzle can the whole picture begin to emerge.

## References

[hbm23188-bib-0001] Alexander DC , Hubbard PL , Hall MG , Moore EA , Ptito M , Parker GJM , Dyrby TB (2010): Orientationally invariant indices of axon diameter and density from diffusion mri. Neuroimage 52:1374–1389. 20580932 10.1016/j.neuroimage.2010.05.043

[hbm23188-bib-0002] Ball G , Srinivasan L , Aljabar P , Counsell SJ , Durighel G , Hajnal JV , Rutherford MA , Edwards AD (2013): Development of cortical microstructure in the preterm human brain. Proc Natl Acad Sci U S A 110:9541–9546. 23696665 10.1073/pnas.1301652110 PMC3677430

[hbm23188-bib-0003] Ball G , Pazderova L , Chew A , Tusor N , Merchant N , Arichi T , Allsop JM , Cowan FM , Edwards AD , Counsell SJ (2015): Thalamocortical connectivity predicts cognition in children born preterm. Cereb Cortex 25:4310–4318. doi:10.1093/cercor/bhu331. 25596587 PMC4816783

[hbm23188-bib-0004] Behrens TEJ , Berg HJ , Jbabdi S , Rushworth MFS , Woolrich MW (2007): Probabilistic diffusion tractography with multiple fibre orientations: What can we gain? Neuroimage 34:144–155. 17070705 10.1016/j.neuroimage.2006.09.018 PMC7116582

[hbm23188-bib-0005] Brody BA , Kinney HC , Kloman AS , Gilles FH (1987): Sequence of central nervous system myelination in human infancy. i. an autopsy study of myelination. J Neuropathol Exp Neurol 46:283–301. 3559630 10.1097/00005072-198705000-00005

[hbm23188-bib-0006] Brown CJ , Miller SP , Booth BG , Andrews S , Chau V , Poskitt KJ , Hamarneh G (2014): Structural network analysis of brain development in young preterm neonates. Neuroimage 101:667–680. 25076107 10.1016/j.neuroimage.2014.07.030

[hbm23188-bib-0007] Chomiak T , Hu B (2009): What is the optimal value of the g‐ratio for myelinated fibers in the rat CNS? a theoretical approach. PLoS One 4:e7754. 19915661 10.1371/journal.pone.0007754 PMC2771903

[hbm23188-bib-0008] Costeloe KL , Hennessy EM , Haider S , Stacey F , Marlow N , Draper ES (2012): Short term outcomes after extreme preterm birth in england: Comparison of two birth cohorts in 1995 and 2006 (the epicure studies). BMJ 345:e7976. 23212881 10.1136/bmj.e7976 PMC3514472

[hbm23188-bib-0009] Eaton‐Rosen Z , Melbourne A , Orasanu E , Cardoso MJ , Modat M , Bainbridge A , Kendall GS , Robertson NJ , Marlow N , Ourselin S (2015): Longitudinal measurement of the developing grey matter in preterm subjects using multi‐modal mri. Neuroimage 111:580–589. 25681570 10.1016/j.neuroimage.2015.02.010

[hbm23188-bib-0010] Hagmann CF , Vita ED , Bainbridge A , Gunny R , Kapetanakis AB , Chong WK , Cady EB , Gadian DG , Robertson NJ (2009): T2 at mr imaging is an objective quantitative measure of cerebral white matter signal intensity abnormality in preterm infants at term‐equivalent age. Radiology 252:209–217. 19561257 10.1148/radiol.2522080589

[hbm23188-bib-0011] He L , Parikh NA (2013): Automated detection of white matter signal abnormality using t2 relaxometry: Application to brain segmentation on term mri in very preterm infants. Neuroimage 64:328–340. 22974556 10.1016/j.neuroimage.2012.08.081 PMC3544934

[hbm23188-bib-0012] Huppi PS , Dubois J (2006): Diffusion tensor imaging of brain development. Semin Fetal Neonatal Med 11:489–497. 16962837 10.1016/j.siny.2006.07.006

[hbm23188-bib-0013] Jones DK , Knsche TR , Turner R (2013): White matter integrity, fiber count, and other fallacies: The do's and don'ts of diffusion mri. Neuroimage 73:239–254. 22846632 10.1016/j.neuroimage.2012.06.081

[hbm23188-bib-0014] Kendall GS , Melbourne A , Johnson S , Price D , Bainbridge A , Gunny R , Huertas‐Ceballos A , Cady EB , Ourselin S , Marlow N , Robertson NJ (2013): White matter naa/cho and cho/cr ratios at mr spectroscopy are predictive of motor outcome in preterm infants. Radiology 271:230–238. 24475798 10.1148/radiol.13122679

[hbm23188-bib-0015] Kreis R , Hofmann L , Kuhlmann B , Boesch C , Bossi E , Hppi PS (2002): Brain metabolite composition during early human brain development as measured by quantitative in vivo 1h magnetic resonance spectroscopy. Magn Reson Med 48:949–958. 12465103 10.1002/mrm.10304

[hbm23188-bib-0016] Kulikova S , Hertz‐Pannier L , Dehaene‐Lambertz G , Buzmakov A , Poupon C , Dubois J (2015): Multi‐parametric evaluation of the white matter maturation. Brain Struct Funct 220:3657–3672. doi:10.1007/s00429-014-0881-y. 25183543 PMC4575699

[hbm23188-bib-0017] Kunz N , Zhang H , Vasung L , O'Brien KR , Assaf Y , Lazeyras F , Alexander DC , Hppi PS (2014): Assessing white matter microstructure of the newborn with multi‐shell diffusion mri and biophysical compartment models. Neuroimage 96:288–299. 24680870 10.1016/j.neuroimage.2014.03.057

[hbm23188-bib-0018] Laule C , Leung E , Li D , Traboulsee A , Patya D , MacKay A , Moore G (2006): Myelin water imaging in multiple sclerosis: Quantitative correlations with histopathology. Multiple Sclerosis 12:747–753. 17263002 10.1177/1352458506070928

[hbm23188-bib-0019] Laule C , Kozlowski P , Leung E , Li DKB , Mackay AL , Moore GRW (2008): Myelin water imaging of multiple sclerosis at 7 t: Correlations with histopathology. Neuroimage 40:1575–1580. 18321730 10.1016/j.neuroimage.2007.12.008

[hbm23188-bib-0020] Maedler B , Drabycz SA , Kolind SH , Whittall KP , MacKay AL (2008): Is diffusion anisotropy an accurate monitor of myelination? correlation of multicomponent t2 relaxation and dif‐ fusion tensor anisotropy in human brain. Magn Reson Imaging 26:874–888. 18524521 10.1016/j.mri.2008.01.047

[hbm23188-bib-0021] McKinstry RC , Mathur A , Miller JH , Ozcan A , Snyder AZ , Schefft GL , Almli CR , Shiran SI , Conturo TE , Neil JJ (2002): Radial organization of developing preterm human cerebral cortex revealed by non‐invasive water diffusion anisotropy MRI. Cereb Cortex 12:1237–1243. 12427675 10.1093/cercor/12.12.1237

[hbm23188-bib-0022] Melbourne A , Cardoso MJ , Kendall GS , Modat M , Robertson NJ , Marlow N , Ourselin S (2013a): AdaPT: An adaptive preterm segmentation algorithm for neonatal brain MRI. Neuroimage 65:97–108. 22906793 10.1016/j.neuroimage.2012.08.009

[hbm23188-bib-0023] Melbourne A , Eaton‐Rosen Z , Bainbridge A , Kendall GS , Cardoso MJ , Robertson NJ , Marlow N , Ourselin S (2013b): Measurement of myelin in the preterm brain: Multi‐compartment diffusion imaging and multi‐component T2 relaxometry. MICCAI 8150: 336–344. 10.1007/978-3-642-40763-5_42 24579158

[hbm23188-bib-0024] Melbourne A , Eaton‐Rosen Z , Vita ED , Bainbridge A , Cardoso MJ , Price D , Cady E , Kendall GS , Robertson NJ , Marlow N , Ourselin S. 2014a Multi‐modal measurement of the myelin‐to‐ axon diameter g‐ratio in preterm‐born neonates and adult controls. MICCAI 8674. Lecture Notes in Computer Science, 268–275. 10.1007/978-3-319-10470-6_34 25485388

[hbm23188-bib-0025] Melbourne A , Kendall GS , Cardoso MJ , Gunny R , Robertson NJ , Marlow N , Ourselin S (2014b): Preterm birth affects the developmental synergy between cortical folding and cortical connectivity observed on multimodal mri. NeuroImage 89:23–34. 24315841 10.1016/j.neuroimage.2013.11.048

[hbm23188-bib-0026] Moffett JR , Ross B , Arun P , Madhavarao CN , Namboodiri AMA (2007): N‐acetylaspartate in the cns: From neurodiagnostics to neurobiology. Prog Neurobiol 81:89–131. 17275978 10.1016/j.pneurobio.2006.12.003 PMC1919520

[hbm23188-bib-0027] Nossin‐Manor R , Card D , Morris D , Noormohamed S , Shroff MM , Whyte HE , Taylor MJ , Sled JG (2013): Quantitative mri in the very preterm brain: Assessing tissue organization and myelination using magnetization transfer, diffusion tensor and t1 imaging. Neuroimage 64:505–516. 22982360 10.1016/j.neuroimage.2012.08.086

[hbm23188-bib-0028] Pandit AS , Robinson E , Aljabar P , Ball G , Gousias IS , Wang Z , Hajnal JV , Rueckert D , Counsell SJ , Montana G , Edwards AD (2014): Whole‐brain mapping of structural connectivity in infants reveals altered connection strength associated with growth and preterm birth. Cereb Cortex 24:2324–2333. 23547135 10.1093/cercor/bht086

[hbm23188-bib-0029] Partridge SC , Mukherjee P , Henry RG , Miller SP , Berman JI , Jin H , Lu Y , Glenn OA , Ferriero DM , Barkovich AJ , Vigneron DB (2004): Diffusion tensor imaging: Serial quantitation of white matter tract maturity in premature newborns. Neuroimage 22:1302–1314. 15219602 10.1016/j.neuroimage.2004.02.038

[hbm23188-bib-0030] Prasloski T , Mdler B , Xiang QS , MacKay A , Jones C (2012): Applications of stimulated echo correction to multicomponent t2 analysis. Magn Reson Med 67:1803–1814. 22012743 10.1002/mrm.23157

[hbm23188-bib-0031] Salami M , Itami C , Tsumoto T , Kimura F (2003): Change of conduction velocity by regional myelination yields constant latency irrespective of distance between thalamus and cortex. Proc Natl Acad Sci U S A 100:6174–6179. 12719546 10.1073/pnas.0937380100 PMC156345

[hbm23188-bib-0032] Stikov N , Perry LM , Mezer A , Rykhlevskaia E , Wandell BA , Pauly JM , Dougherty RF (2011): Bound pool fractions complement diffusion measures to describe white matter micro and macrostructure. Neuroimage 54:1112–1121. 20828622 10.1016/j.neuroimage.2010.08.068 PMC2997845

[hbm23188-bib-0033] Volpe JJ (2009): Brain injury in premature infants: A complex amalgam of destructive and devel‐ opmental disturbances. Lancet Neurol 8:110–124. 19081519 10.1016/S1474-4422(08)70294-1 PMC2707149

[hbm23188-bib-0034] Woodward LJ , Anderson PJ , Austin NC , Howard K , Inder TE (2006): Neonatal MRI to predict neurodevelopmental outcomes in preterm infants. N Engl J Med 355:685–694. 16914704 10.1056/NEJMoa053792

[hbm23188-bib-0035] Zhang H , Schneider T , Wheeler‐Kingshott CA , Alexander DC (2012): Noddi: Practical in vivo neurite orientation dispersion and density imaging of the human brain. Neuroimage 61:1000–1016. 22484410 10.1016/j.neuroimage.2012.03.072

